# Supplementary material for: Bacteria-mediated green synthesis of silver nanoparticles and their antifungal potentials against Aspergillus flavus
Source: PLoS One. 2024 Mar 25;19(3):e0297870. doi: 10.1371/journal.pone.0297870 (PMC10962810; doi:10.1371/journal.pone.0297870)
Supplement: S1 File — (PDF) [file pone.0297870.s001.pdf]

Treat ment No.	Treatment	Antagonism	% Growth Inhibition of A. flavus
**T1**	PDA (20 ml) + Pathogen* (Control)	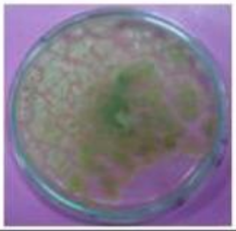	**0.00**
**T2**	PDA (20 ml) + Fungus + *B. subtilis* JND-KHGn-29-A (live antagonist)	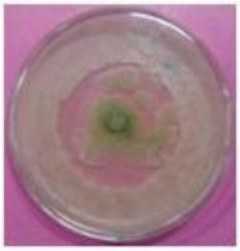	**57.05**
**T3**	PDA (20 ml) containing 1 ml *B. subtilis* (2.7×10^7 cfu)+ Pathogen	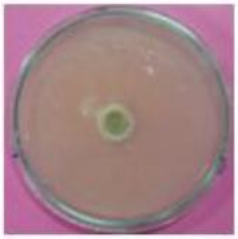	**72.53**
**T4**	PDA + 200 ul nanoparticles (NPs) + Pathogen	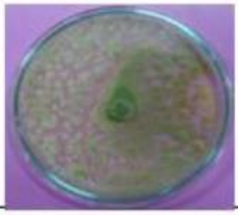	**67.03**
**T5**	PDA + 500 ul NPs + Pathogen	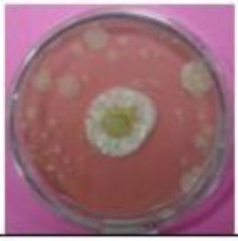	**68.53**
**T6**	PDA + 1ml NPs (19:1) + Pathogen	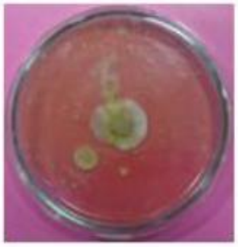	**82.53**

T7	PDA + 2ml NPs (18:2) Pathogen	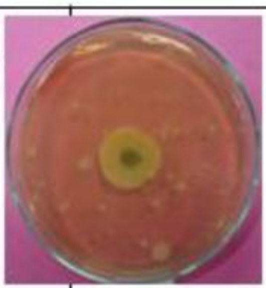	70.53
T8	T8= PDA + 3ml NPs (17:3) + Pathogen	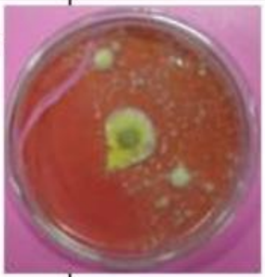	55.00
T9	PDA + 4ml NPs (16:4 Pathogen	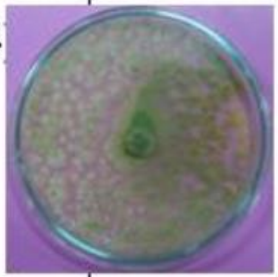	0.00
T10	PDA + 5ml Nano (15: + Pathogen	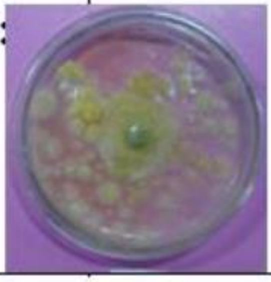	0.00

Figure S1: *In-vitro* study to derive minimum inhibition of green bacterial nanoparticles for inhibition of toxic *A. flavus* JAM-JKB-BHAGG20. **A. flavus* JAM-JKB-BHA-GG20

Variety	Kernel	Seed	After 5 days ($\mu g.kg^{-1}$)	After 10 days ($\mu g.kg^{-1}$)
1. GG-2	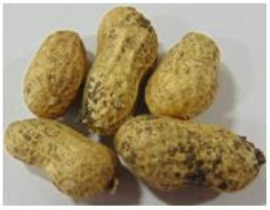	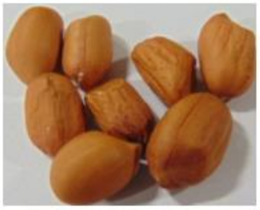	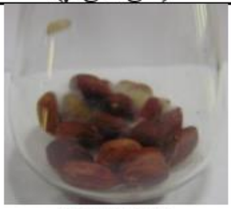 (232.20)	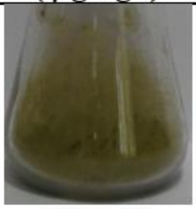 2736.50
2. GG-5	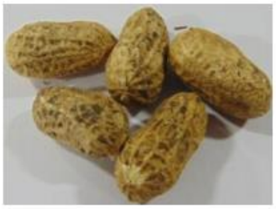	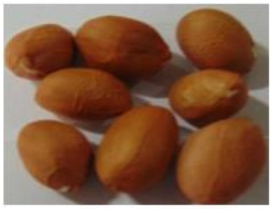	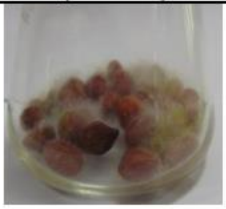 330.09	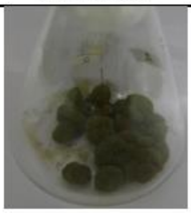 1847.98
3. GG-6	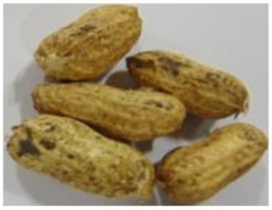	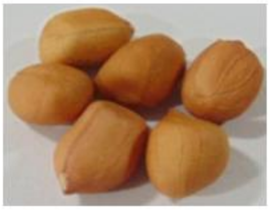	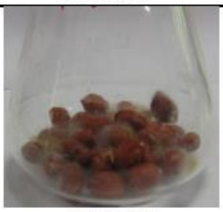 170.67	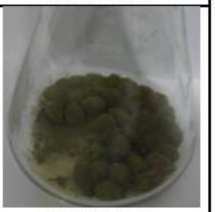 1403.35
4. GG-7	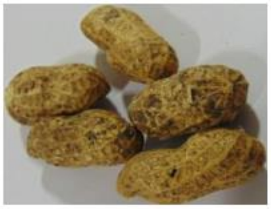	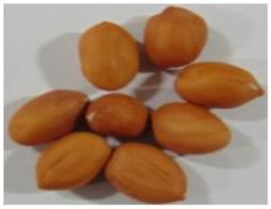	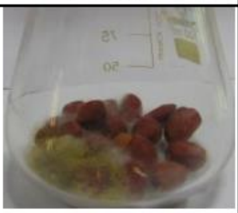 294.56	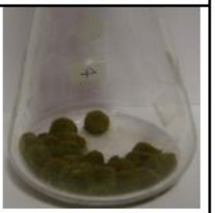 1340.67
5. GG-8	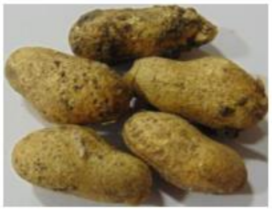	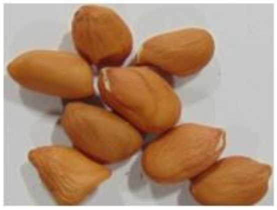	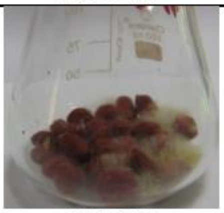 235.92	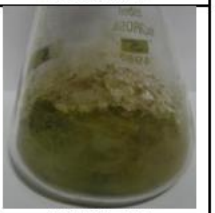 4010.47
6. GG-9	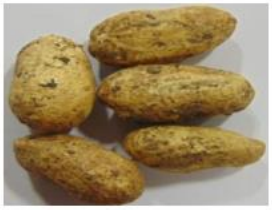	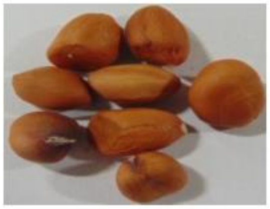	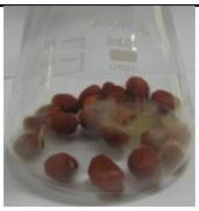 169.46	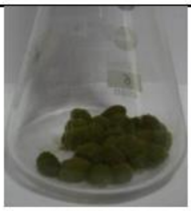 4925.41
7. SB-XI	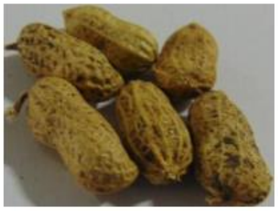	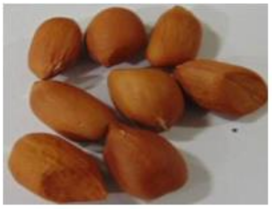	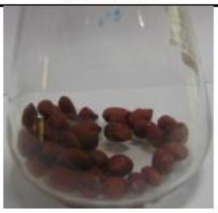 **101.10**	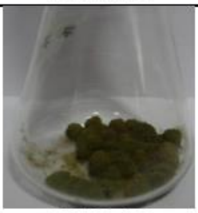 6010.53

Variety	Kernel	Seed	After 5 days ($\mu g.kg^{-1}$)	After 10 days ($\mu g.kg^{-1}$)
8. GAUG-10	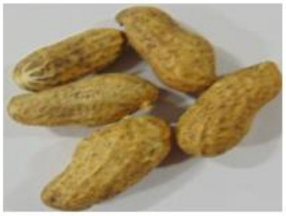	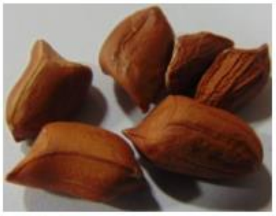	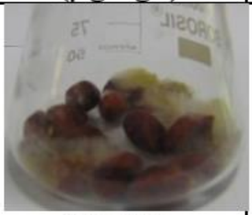 330.35	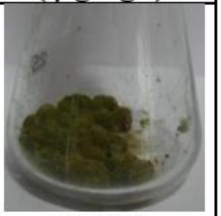 854.33
9. GG-11	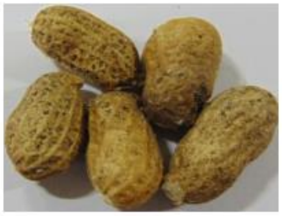	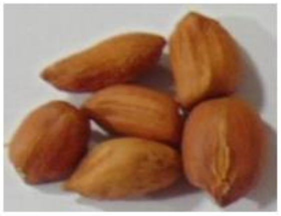	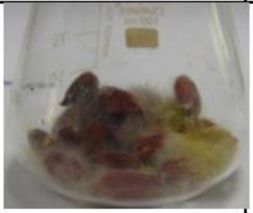 239.59	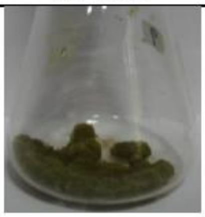 7268.15
10. GG-12	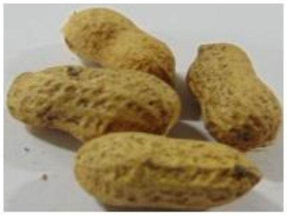	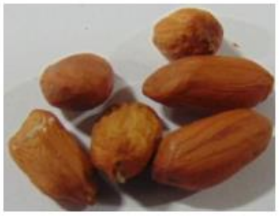	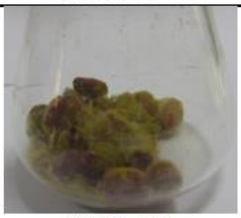 4049.07	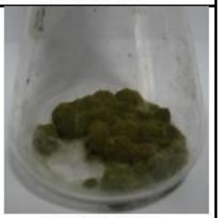 3672.00
11. GG-13	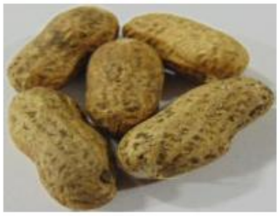	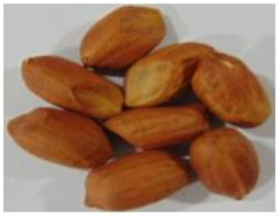	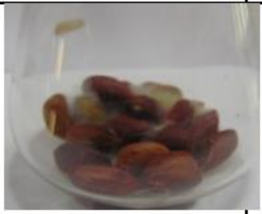 279.95	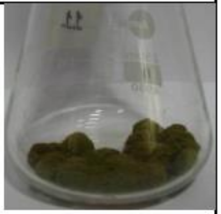 14505.12
12. GG-14	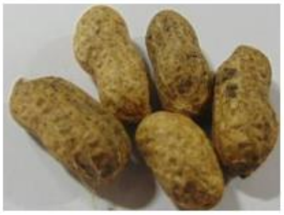	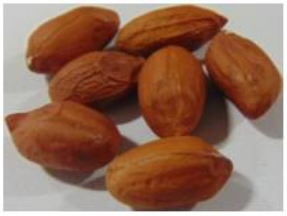	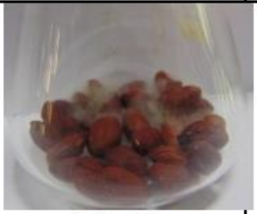 **103.63**	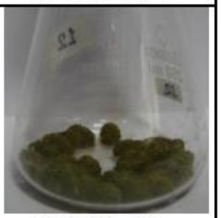 10719.30
13. GJG-17	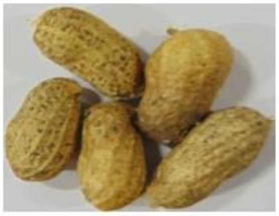	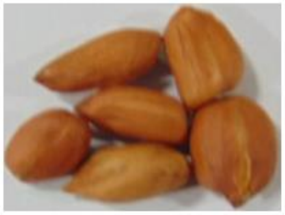	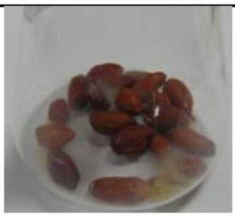 166.36	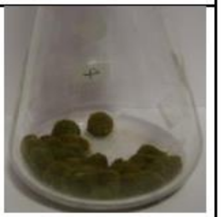 **550.99**
14. GJG-18	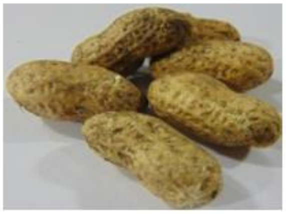	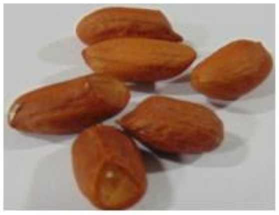	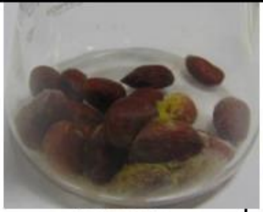 115.25	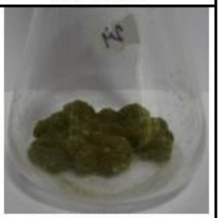 23984.92

Variety	Kernel	Seed	After 5 days ($\mu g.kg^{-1}$)	After 10 days ($\mu g.kg^{-1}$)
15. GJG-19	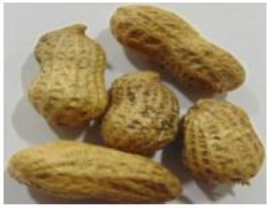	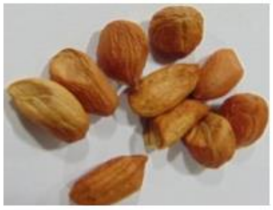	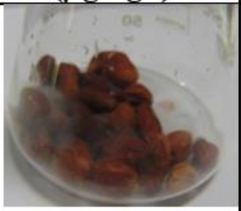 **100.80**	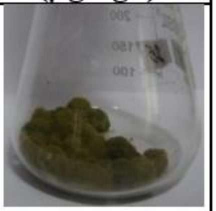 28655.14
16. GG-15	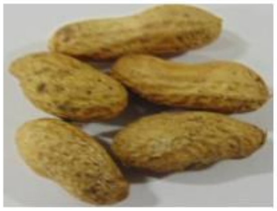	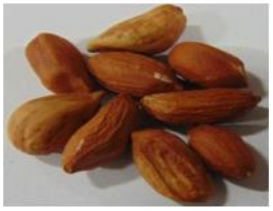	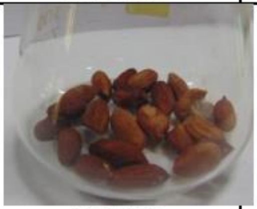 157.58	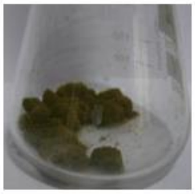 21882.85
17. GG-20	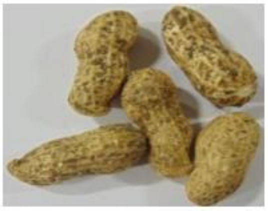	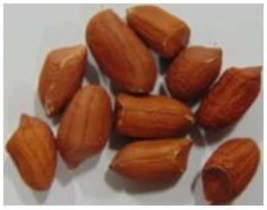	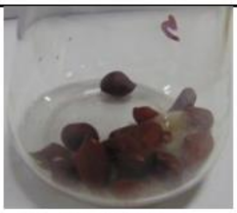 284.23	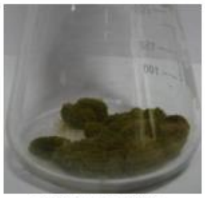 23418.00
18. GG-21	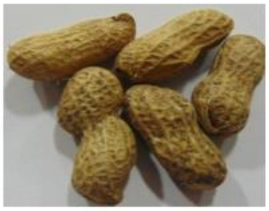	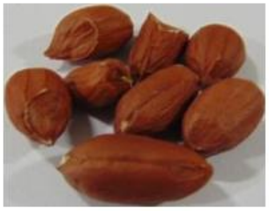	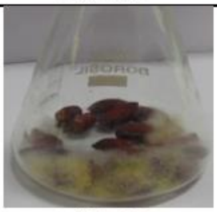 779.23	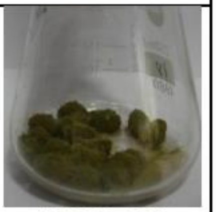 15650.27
19. GG-22	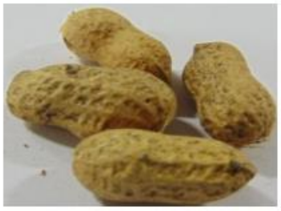	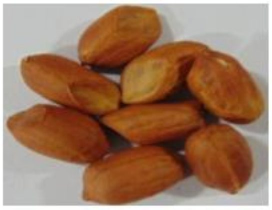	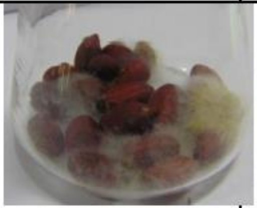 348.25	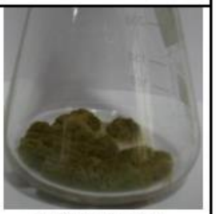 **35584.65**
20. TG-26	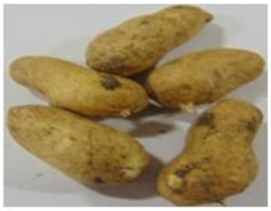	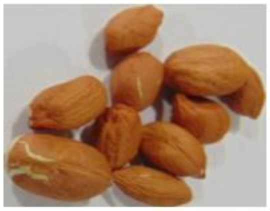	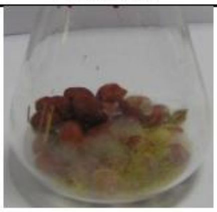 145.85	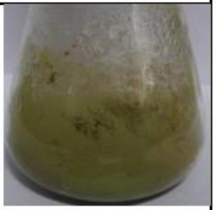 5553.31
21. HPS-1	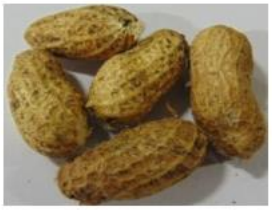	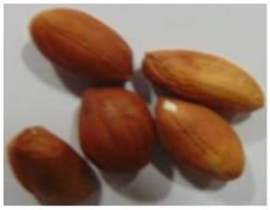	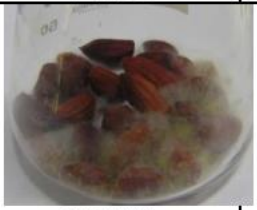 222.71	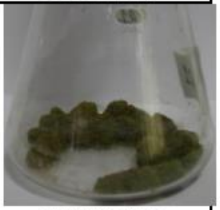 15172.54

Variety	Kernel	Seed	After 5 days (µg.kg^{-1})	After 10 days (µg.kg^{-1})
22. TG-37	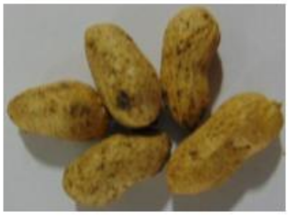	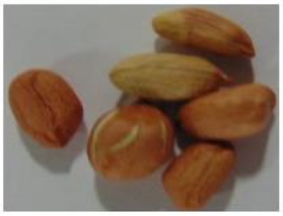	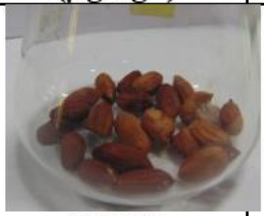 4027.51	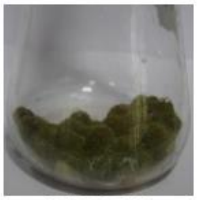 4690.22
23. TG-45	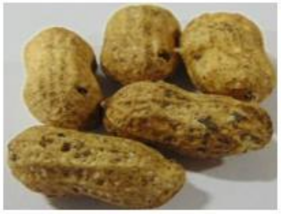	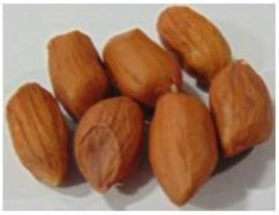	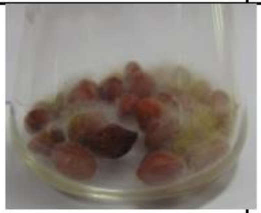 5603.59	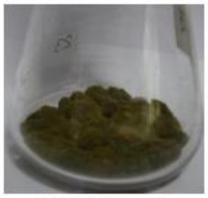 18751.68
24. TG-51	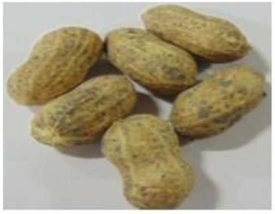	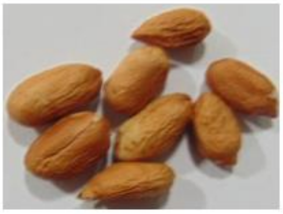	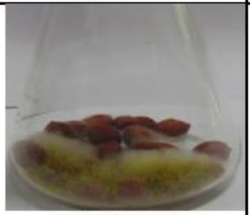 **6111.17**	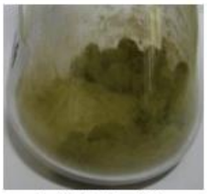 14577.48
25. TPG-41	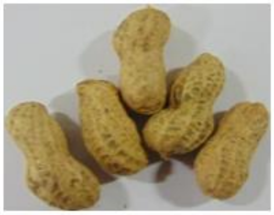	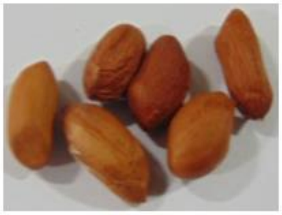	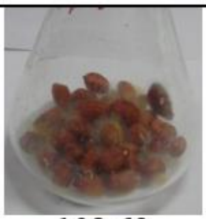 108.69	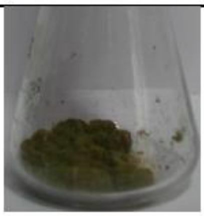 15859.21

* values in paranthesis indicates total aflatoxin detection in ppb

Figure S2: *In-vitro* screening of 25 groundnut varieties for aflatoxin production by most toxic and virulent *A. flavus* JAM-JKB-BHA-GG20 at different time interval
