# Supplementary material for: Bacteria-mediated green synthesis of silver nanoparticles and their antifungal potentials against Aspergillus flavus
Source: PLoS One. 2024 Mar 25;19(3):e0297870. doi: 10.1371/journal.pone.0297870 (PMC10962810; doi:10.1371/journal.pone.0297870)
Supplement: Attachment — Submitted filename: Response to Reviewers.docx [file pone.0297870.s002.docx]

**Response to Reviewers Comments**

**Academic Editor**

1. Financial statement has been modified in the revised manuscript.

2. Data availability statement has been modified in the revised manuscript.

3. Captions for the supporting information have been provided in the revised paper.

4. Materials and method section has been moved before the result section in the revised version of paper.

5. Brief description for isolation of *B. subtilis* has been added in the revised draft of paper.

6. All references are properly cited in the revised paper.

7. Statement for replicates has been included in the revised paper.

8. Since, the manuscript involves most of the analysis part through various instrumentation it does not required appropriately and rigorously statistical analysis.

**Reviewer 1**

1. We apologize for the typographical errors in Table 4 and Figure 6. The same has been corrected in the revised paper by mentioning G22 as susceptible variety.

2. We agree with the learn reviewer that TG-51 and GG-22 were found to be the most sensitive groundnut varieties after 5 and 10 days of infection by LC-MS QTOF when 25 different groundnut varieties were screened using the most toxic *A. flavus* isolate JAM- JKB-BHA-GG20 respectively. The most susceptible groundnut cultivar, designated as GG-22, was tested in this research. Because less aflatoxin (1651.15 g.kg-1) was observed, treatment T8 (Seed + Pathogen + 2 m1 AgNPs) was determined to be much more effective. Hence, G-22 was designated as most susceptible varieties and used for further studies.

3. The details of isolation of *Bacillus* and *Aspergillus* species have been provided in the revised paper.

4. The culture flask means the conical flasks which were used for experiments.

5. Culture supernatant antagonist refers to a substance obtained from the liquid portion of bacterial culture which has antagonist effect by inhibiting the growth of *Aspergillus* which indicates the interaction and dynamics within the microbial communities.

6. Spelling of Groundnut varieties has been corrected in Figure 5.

**Reviewer 2**

1. We agree with the learned reviewer and the quality of English language have been improved by taking the help of person who is expert in English language.

2. As suggested by learned reviewer a brief description for synthesis of silver particles have been inserted in the revised manuscript.

3. *Bacillus subtilis* posses the enzyme nitrate reductase enzyme which helps in reduction and stabilization of silver nanoparticles. This is common mechanism. Synthesis of silver nanoparticles was confirmed by Utilizing UV-Visible spectroscopy, PSA and Zeta Potential Analysis, Scanning Electron Microscopy, and Fourier Transform Infrared Spectroscopy. The same has been clearly mentioned in the revised paper.

4. Since, the manuscript involves most of the analysis part through various instrumentation it does not required appropriately and rigorously statistical analysis. However, all the important statistical analysis was utilized to prove the significance of study. The statistical analysis are in clearly indicates in respective tables and figures.

5. As recommended by reviewer discussion has been modified in the revised paper.

6. The yield of nanoparticles was in the range of 50 to 67 nm.

7. We agree with the learn reviewer and the paragraph superiorities of microbial synthesis of nanoparticles over traditional physical and chemical synthesis has been added in the revised manuscript.

8. Abbreviations are put later than the actual word in the revised paper as recommended by learn reviewer.

9. All spellings, grammatical and typo errors are corrected in the revised paper by taking the help of person who is native in English.

10. The purity and grade of chemical has been added in the revised paper.

11. We agree with the reviewer however, all the figures are modified as per the PACE software tools to meet the requirement of journals and also improved the quality of figures.

12. We totally agree with the learn reviewer however, FITR is shown in the range of 1800-1100 to demonstrate the best possible results by referring standard references.

13. Sub-headings are modified in the revised paper.

14. Abbreviations are removed from abstract section in revised paper.

25. The word ‘Anti-fungicidal activity’ was replace by ‘Anti-fungal activity’ in revised paper.

16. Figure 4 shows the correlation among each other and therefore it is shown together and modified through PACE to meet the journal requirements.

17. We agree with the learn reviewer however, no growth was observed for fungus across the centre well which confirmed that the growth was inhibited by more than 70%.

18. Justification for cost effective of silver nanoparticles has been provided in the revised paper under discussion section.

19. Culture was centrifuged to remove the impurities in order to obtain pure nanoparticles and is clearly mentioned in paper.

20. Bacterial strains were discarded by autoclaving them by referring safety laboratory protocols.

21. Drying process is not included in this study as we follow other standard reference published previously and hence is not mentioned in the paper.

22. We agree that the *In-vitro* fungicidal activity of different concentrations of silver nanoparticles can be included (Culture plates) as visual evidence and is provided as supplementary file for clear understanding. Also we have to follow journal guidelines to reduce number of tables and figures.

**Other Corrections**

1. All the typographical errors, minor corrections, etc. are highlighted with the track change in the revised manuscript.
